# Monoclonal antibodies for the therapy of cancer

**DOI:** 10.1186/1753-6561-8-S4-O6

**Published:** 2014-10-01

**Authors:** Andrew Simpson, Otavia Caballero

**Affiliations:** 1 Orygen Biotecnologia S.A, São Paulo, SP, Brazil; 2 Ludwig Institute, São Paulo, SP, Brazil

## Introduction

### The introduction of monoclonal antibodies for the treatment of cancer

Monoclonal Antibodies (mAbs) comprise a class of therapeutic biologics that has been increasingly used over the last decades. The concept of using antibodies to selectively target tumors was proposed by Paul Ehrlich over a century ago [1]. The development of the hybridoma technology in 1975 enabled the production of monoclonal antibodies, which contain uniform variable regions and are thus specific for a single epitope. They are immunoglobulin molecules secreted from a population of identical cells and are homogeneous in structure and binding specificity. Their specificity for the target, together with the fact that they are relatively well tolerated and have a long half-life has contributed to their success in drug development.

Antibodies can trigger direct effects on tumor growth causing apoptosis or inhibition of proliferation, they can also mediate immune effector functions. The first antibodies used in the clinic were of murine origin. Due to their immunogenicity in humans and poor ability to induce human immune effector responses, they exhibited limited clinical applicability. Chimeric, humanized and fully human monoclonal antibodies have now been developed to address these problems. Chimeric antibodies are encoded by genes from more than one species, usually with antigen-binding regions from mouse genes and constant regions from human genes, while humanized antibodies are genetically engineered mouse antibody in which the protein sequence has been modified to mimic that of human antibodies [1]. Antibodies can be subdivided into two distinct functional units: the fragment of antigen binding (Fab) and the constant fragment (Fc). The Fab contains the variable region, which consists of three hypervariable complementarity-determining regions (CDRs) that form the antigen binding site of the antibody and confer antigen specificity. The Fc can bind to immune effector cells and complement that can both mediate antibody directed immune killing.

### Mechanism of action of monoclonal antibodies for the treatment of cancer

#### Altering signal transduction in the downstream intracellular pathways

Cancer cells express various cell surface receptors that activate intracellular pathways leading to growth. Amongst these, EGFR or ErbB1, ErbB2 or HER-2/Neu, HER-3 and HER-4 are of the same family and are overexpressed in epithelial malignancies originating from the colon, breast, lung and head and neck resulting in rapidly proliferating disease and increased metastatic potential. Anti-EGFR antibodies bind to the receptor domain of the EGFR receptor inhibiting the downstream activation of the receptor and increasing receptor internalization. These antibodies can inhibit the cancer cell cycle leading to apoptosis. In addition, the combination of antibodies with chemotherapy enhances the activity of chemotherapy. AntiHER-2 antibodies promote receptor internalization and cell cycle arrest. Anti-HER2 antibodies can also block heterodimer formation between HER-2 and HER-3 or HER-4 providing an additional mode of action [2].

#### Antibody-dependent cytotoxicity (ADCC)

This mechanism results in the immune-mediated destruction of the cancer cells that are coated by antibodies. The effector cells in the antibody-dependent cytotoxicity include macrophages, NK cells and neutrophils. ADCC depends on the Fc portion of the antibody that binds a Fc gamma receptor (FcgR) on the effector cells. ADCC occurs when the Fab and Fc portions of the mAb engage both tumor cell antigen and an activating FcgR, respectively, thus creating a bridge from the tumour cell to the effector cell. Target cell recognition is then coupled to a lytic attack on the target cell mounted by effector cells. Several studies have established the importance of FcgR interactions for the *in vivo *antitumor effects of certain monoclonal antibodies in murine models and clinical trials. The antitumor activities of trastuzumab and rituximab have been shown to be lower in FcgR -deficient mice than wild-type mice, for example [2]. The role of FcgR in the antitumor response has been further supported by the finding that polymorphisms in genes encoding FcgR result in differential response rates to therapeutic monoclonal antibodies [3].

#### Complement-mediated cytotoxicity (CDC)

CDC results from a cytolytic cascade mediated by a series of complement proteins, resulting in lysis of the antibody-bound cell [2]. Antibody ability to bind complement varies with the Ig isotypes. Interactions with IgM, IgG1, and IgG3 are strong while IgG2 is a poor inductor and IgG4 is devoid of complement activation functions. CDC has been shown to be an important mechanism of action of rituximab and depletion of complement in mouse models led to the complete loss of activity of rituximab [4].

#### Soluble ligand neutralization

Antibodies can bind to circulating proteins and interfere with their ability to find their targets to help facilitate growth of the tumors. One important example of this mechanism is bevacizumab which is a fully humanized monoclonal antibody against VEGF-A. Bevacizumab binds and inactivates the biological activity of VEGF-A, inhibiting angiogenesis and thus, tumor growth and proliferation [5]. Siltuximab is another monoclonal antibody that works by ligand neutralization. It binds to IL-6 thus inhibiting the further action of IL-6 on tumor growth [6].

#### Cytotoxic drug delivery

Cytotoxic agents can be linked to tumor targeted monoclonal antibodies with the aim of delivering them specifically to the tumors cells [7]. This approach limits systemic side effects. Two exciting examples of this technology are trastuzumab-DM1 T-DM1, a HER2 directed antibody drug conjugate, and brentuximab vedotin, a CD30 directed antibody drug conjugate [2].

##### The first generation

Several antibodies have been approved by the FDA for the treatment of a variety of solid tumors and hematological malignancies (Table 1). Therapeutic antibodies targeting the EGFR (epidermal growth factor receptor) and VEGF (vascular endothelial growth factor) have been successfully used for treatment of patients with a variety of solid tumors. Trastuzumab has also been successfully used in patients with either immunohistochemical staining or fluorescence in situ hybridization positive for HER-2 expression. In the hematologic tumors, the most successful target has been CD20 on B-lymphocyte cells [2,8]. In Brazil, biosimilar antibodies are being developed to provide improved access to the first generation of anti-cancer antibodies. Those being developed include antibodies against CD20, Her2, EGFR and VEGF-A.

**Table 1:** Monoclonal antibodies approved for clinical use in oncology

Antibody name	Target	Antibody format	Application
**Cetuximab**	EGFR	Chimeric	Colorectal, breast and lung cancer

**Panitumumab**	EGFR	Human	Colorectal cancer

**Nimotuzumab**	EGFR	Humanized	Head and neck cancer

**Rituximab**	CD20	Chimeric	Non-Hodgkin lymphoma

**Trastuzumab**	HER2	Humanized	Breast cancer

**Alemtuzumab**	CD52	Humanized	Chronic lymphocytic leukemia

**Bevacizumab**	VEGFA	Humanized	Colorectal and lung cancer

**Ofatumumab**	CD20	Human	Chronic lymphocytic leukemia

**Ipilimumab**	CTLA-4	Human	Metastatic melanoma

**Pertuzumab**	HER2	Humanized	Breast cancer

**Denosumab**	RANK Ligand	Human	Solid tumor bony metastases

**Brentuximab vedotin**	CD30	Chimeric	Hodgkin's or systemic anaplastic large cell lymphoma

**Gemtuzumabozogamicin**	CD33	Humanized	Acute myelogenous leukemia

**90Y-Ibritumomab tiuxetan**	CD20	Mouse	Low grade or transformed B cell non-Hodgkin's lymphoma

**Tositumomaband 131I-tositumomab**	CD20	Mouse	Lymphoma

##### The second generation

There are multiple therapeutic and diagnostic antibodies that are currently under testing or development [8]. The current pipeline of anticancer mAbs in clinical studies includes more than 100 candidates. The canonical bivalent, monospecific, full-length IgG molecule is representative of about half the anticancer mAbs in the pipeline [9]. The remaining are noncanonical candidates that are conjugated to drugs or radiolabels, multispecific molecules or antibodies engineered for increased functionality, and antibody fragments. Advances in protein- and glyco-engineering are now enabling the production of next-generation mAbs that are potentially more efficacious compared to the first-generation versions. Although the use of antibodies to guide drugs to a specific target has been explored for over 30 years, advances in the knowledge of linker and drug properties, and antibody engineering, design and selection, have led to the development of a new generation of molecules that are demonstrating promising clinical results [9]. One example is trastuzumab emtansine, a humanized anti-HER2 antibody conjugated to DM1 that is undergoing evaluation in Phase III studies of breast cancer patients [7]. Bispecific antibodies, as the name suggests, are designed to bind two different targets. Modifications in protein sequence have been shown to extend antibody half-life and afucosylated mAbs are known to have enhanced effector functions [9]. Most anticancer mAbs kill cells directly by binding to an antigen associated with a tumor cell and inducing cell death via effector functions, cytotoxic payloads or blockade of signals required for growth. However, in the last few years, antibodies that present indirect mechanisms of action, such as the activation of the immune system to combat tumors, have been developed. Ipilimumab, a first in class T-cell potentiator was approved for treatment of metastatic melanoma and represents the first therapy demonstrated to improve overall survival in melanoma [10]. Other advances illustrate how new insights into the structure and function of receptors allow them to be targeted in novel ways, with expected improvements in the therapeutic efficacy. EGFR is the target for three marketed anticancer mAbs (cetuximab, panitumumab and nimotuzumab) (Table 1). Recently, monoclonal antibody 806 (mAb806) was shown to target a conformational epitope exposed on wild-type EGFR when it is overexpressed on tumor cells or induced by the presence of oncogenic mutations such as EGFRvIII. The mechanism of action of mAb806, which allows for EGFR inhibition without normal tissue toxicity, creates opportunities for combination therapy and strongly suggests mAb806 will be a superior targeted delivery system for antitumor agents. This concept of conformational epitope targeting by antibodies reflects an underlying interplay between the structure and biology of different conformational forms of the EGFR family [10].

## Conclusions

The number of monoclonal antibodies that have been approved or are in development for cancer treatment has been steadily increasing over the last years. Because of their high target specificity, generally low toxicity and the ability to activate the immune system, the use of therapeutic antibodies for cancer treatment is very promising. They currently provide clinical benefit to patients with cancer and have been established as 'standard of care' agents for several tumor types. Advances in the identification and validation of new targets, manipulation of tumor-host microenvironment interactions, and optimization of antibody structure to promote anti-tumor immune responses will generate many new treatments for cancer over the next decade.
